# Analyses of Final Trends and Comorbidity of Eosinophilic Granulomatosis with Polyangiitis in Patients Hospitalized in Poland in 2014–2023

**DOI:** 10.3390/jcm14248950

**Published:** 2025-12-18

**Authors:** Aleksandra Hus, Krzysztof Kanecki, Katarzyna Lewtak, Paweł Goryński, Małgorzata Wisłowska

**Affiliations:** 1 Department of Rheumatology, National Institute of Geriatrics, Rheumatology and Rehabilitation, Spartańska 1 Street, 02-637 Warsaw, Poland; aleksandra.hus@spartanska.pl; 2 Department of Social Medicine and Public Health, Medical University of Warsaw, Żwirki i Wigury 61 Street, 02-091 Warsaw, Poland; 3 National Institute of Public Health NIH—National Research Institute, Chocimska 24 Street, 00-791 Warsaw, Poland; katarzyna.lewtak@wum.edu.pl; 4 Centre of Postgraduate Medical Education, Kleczewska 61-63 Street, 01-826 Warsaw, Poland

**Keywords:** ANCA-associated: vasculitis, systemic eosinophilic vasculitis, population-based registry, prevalence, multimorbidity, cardiometabolic complications, airway involvement

## Abstract

**Background**: Eosinophilic granulomatosis with polyangiitis (EGPA) is a rare vasculitis, and contemporary population data from Central and Eastern Europe are limited. **Aim**: To describe hospital-based incidence, patient characteristics and comorbidities among EGPA hospitalizations in Poland (2014–2023), including differences by age, sex and place of residence. **Methods**: This retrospective, population-based study used nationwide hospital discharge records with an EGPA diagnosis. First EGPA-coded hospitalizations were used to estimate annual incidence per 1,000,000 inhabitants. Demographics, duration of stay and accompanying comorbidities (cardiovascular disease, pulmonary disease, and asthma) were analyzed for all EGPA hospitalizations. Place of residence was classified as urban or rural. Group differences and temporal trends were assessed using appropriate parametric and non-parametric tests and regression models, with a two-sided *p* value < 0.05 considered statistically significant. **Results**: Between 2014 and 2023, 911 patients had a first EGPA-coded hospitalisation in Poland, corresponding to a mean annual hospital-based incidence of 2.38 per 1,000,000 inhabitants (range 1.28–3.38); incidence declined significantly from 2014 to 2019 (*p* < 0.001) and was disrupted during the COVID-19 period. Overall, 3524 EGPA hospitalisations were recorded, and women were more frequently hospitalised than men (54.5% vs. 45.2%; *p* < 0.001). Mean age at hospitalisation increased over time, with patients in 2023 being about 5–6 years older than in 2014 (*p* ≤ 0.009). Median length of stay was 8 days for first admissions and 5 days for all EGPA stays and shortened significantly over the study period (*p* < 0.001). Cardiovascular disease, pulmonary disease and asthma were present in 23.6%, 35.3% and 32.3% of patients, respectively. Cardiovascular disease was more common in men and in rural residents (both *p* < 0.001) and was associated with older age (*p* < 0.001), whereas pulmonary disease was associated with younger age (*p* < 0.001). Among women, the proportions with pulmonary disease and asthma decreased over time (*p* = 0.009 and *p* = 0.025). **Conclusions**: EGPA in Poland is rare, with hospital-based incidence comparable to other European and Asian populations. The hospitalized EGPA population is aging and cardiovascular comorbidity is increasingly prominent, especially in older and rural patients, while recorded pulmonary disease and asthma in women are decreasing.

## 1. Introduction

Eosinophilic granulomatosis with polyangiitis (EGPA) is a rare systemic vasculitis characterized by necrotizing inflammation of small- to medium-sized vessels associated with eosinophilia and affecting primarily the respiratory tract [1,2]. It is driven by a combination of type 2 helper T-cell-mediated eosinophilic inflammation, cytokines such as interleukin-5, and, in a subset of patients, antineutrophil cytoplasmic antibodies (ANCAs), which together contribute to vascular and tissue injury [1,2,3]. Standard treatment is based on systemic glucocorticoids, often combined with conventional immunosuppressive agents, and in more severe or relapsing disease increasingly includes biologic therapies targeting type 2 inflammation, such as anti-IL-5 monoclonal antibodies (e.g., mepolizumab) [3,4]. Despite these advances, EGPA remains associated with substantial morbidity and risk of organ damage [3,5,6,7].

EGPA was first described by Jacob Churg and Lotte Strauss in 1951, who developed the definition and diagnosis based on the presence of three key features in human autopsies: eosinophilic infiltration, necrotizing vasculitis of small- and medium-sized vessels, and extravascular granuloma formation [1,2]. Over time, the disease started to be recognised by repeatable clinical patterns, namely bronchial asthma, blood eosinophilia of more than 1500 eosinophils/µL and vasculitis involving at least two extrapulmonary organs [1,2]. The diagnostic and classification criteria have changed over time and were updated and validated in 2022 by the American College of Rheumatology and the European Alliance of Associations for Rheumatology (ACR/EULAR). The current classification criteria include seven items: maximum eosinophil count >1 × 10^9^/L, obstructive airway disease, nasal polyps, myeloperoxidase-ANCA (MPO-ANCA) or proteinase 3-ANCA (PR3-ANCA) positivity, extravascular eosinophil-predominant inflammation, mononeuritis multiplex/motor neuropathy not due to radiculopathy, and hematuria [2,3].

Contemporary population-based studies and systematic reviews consistently underline that EGPA is a rare condition despite advances in diagnostics and classification validation. The estimated incidence typically ranges from 1 to 4 cases per 1,000,000 person-years, and prevalence from about 10 to 40 cases per 1,000,000 inhabitants in European populations [5,7,8,9]. The estimated prevalence of the disease worldwide is approximately 1 to 3 per 1,000,000 adults [2,7]. Although global and European EGPA incidence and prevalence are low, the associated disease burden is substantial as the nature of the disease is severe and relapsing. The rising prevalence against a background of stable incidence influences healthcare capacity. EGPA-related healthcare resource use (HCRU) is burdened by frequent inpatient admissions and emergency department visits, reported for 17–42% and 25–42% of patients, respectively [5,8]. Persistent oral glucocorticoid exposure in the year after diagnosis is also an important driver of HCRU and treatment-related morbidity [6,8].

While epidemiological data are essential for understanding the occurrence of EGPA, they do not fully capture the complex clinical burden of the disease. The management of EGPA is often related to a spectrum of comorbidities directly linked to both the disease’s progression and the consequences of its long-term immunosuppressive treatment [5,6,10]. Cardiovascular complications stand as a leading cause of mortality and include vasculitis, atherosclerosis and heart failure [11,12,13]. Furthermore, high cumulative doses of glucocorticoids predispose patients to osteoporosis and fractures, diabetes mellitus, cataracts, glaucoma, infections, cardiovascular diseases and psychosis [6]. Immunosuppression also increases susceptibility to recurrent and severe infections and malignancy [10], while the chronic, relapsing nature of the disease contributes to a high rate of depression and anxiety.

In Poland, a previous nationwide study based on the hospital morbidity database for 2008–2013 estimated an average annual EGPA incidence of 1.5 per 1,000,000 (95% CI 1.2–1.8) and a point prevalence of 8.8 per 1,000,000 at the end of 2013, with higher incidence in more urban regions [14]. However, epidemiological data after 2013 are lacking, and no national analysis has assessed temporal trends in incidence and hospitalisation rates across the subsequent decade, including the period of the COVID-19 pandemic. Moreover, there are limited data about the age and sex distribution and comorbidity profile among EGPA patients in Poland.

Therefore, we analysed the national hospital morbidity database of EGPA in Poland over the period 2014–2023. The primary objectives were to (i) estimate hospital-based occurrence of EGPA, including annual incidence of first EGPA-coded hospitalisations and annual rates of all EGPA-coded hospitalisations; (ii) assess temporal trends in these measures; (iii) characterise the age and sex distribution of hospitalised patients and its changes over time; (iv) describe the burden and trends of comorbidities, including cardiovascular disease, pulmonary disease and asthma; and (v) evaluate duration of EGPA-related hospitalisations.

## 2. Methods

### 2.1. Data Source and Study Design

The study was approved by the institutional Ethics Committee (No. KBT-8/4/2025) and conducted in accordance with the Declaration of Helsinki. This was a retrospective, nationwide, population-based study using hospital discharge records with a diagnosis of eosinophilic granulomatosis with polyangiitis (EGPA) obtained from the Polish National Institute of Public Health for the years 2014–2023. The database covers all hospitalizations in Poland. Information on place of residence was extracted from the registry and categorized according to the administrative classification as urban (city) or rural (village).

### 2.2. Study Population

The study population consisted of all hospitalizations in Poland from 1 January 2014 to 31 December 2023 during which the ICD-10 (International Classification of Diseases) code M30 (The eosinophilic granulomatosis with polyangiitis) appeared for the first time. The inclusion criteria were:

Presence of the ICD-10 code M30 as either the principal or an additional diagnosis;
Hospital admission recorded within the study period (2014–2023);
The ICD-10 code M30 appearing for the first time during the given hospitalization.

For the estimation of incidence, only first EGPA-coded hospitalizations for each patient during the study period were used. All EGPA-coded hospitalizations were included in analyses of age, sex, place of residence, duration of stay and comorbidities.

### 2.3. Variables and Definitions

For each hospitalization we extracted data on age, sex, calendar year of admission, length of hospital stay and place of residence (urban vs. rural). Comorbidities of interest included cardiovascular disease, pulmonary disease and asthma, which were identified based on relevant ICD-10 codes recorded in any diagnosis field.

Annual incidence rates of EGPA were calculated as the number of patients with a first EGPA-coded hospitalization in a given year divided by the total population of Poland in that year and expressed per 1,000,000 person-years. Prevalence was estimated as the number of patients alive with a recorded EGPA diagnosis on the last day of follow-up divided by the total population and expressed per 1,000,000 inhabitants. Incidence and prevalence estimates were accompanied by 95% confidence intervals (CIs).

### 2.4. Statistical Analysis

All analyses were performed using standard statistical software. A two-sided *p*-value < 0.05 was considered statistically significant. Continuous variables were summarized as mean ± standard deviation (SD) or median with interquartile range (IQR), depending on their distribution, and categorical variables as counts and percentages. Analyses were primarily descriptive and exploratory, reflecting the set of variables available in the nationwide hospital discharge registry.

Comparisons between women and men, and between urban and rural residents, for continuous variables (e.g., age, duration of hospitalization) were performed using the independent-samples *t*-test or, in the case of non-normal distributions, the Mann–Whitney U test. Differences in categorical variables (e.g., presence of cardiovascular disease, pulmonary disease or asthma; proportion of women; proportion of urban vs. rural residents) between groups were assessed using the χ^2^ test of independence or Fisher’s exact test, as appropriate.

Differences in age across calendar years were evaluated using one-way analysis of variance (ANOVA) or the Kruskal–Wallis test, followed by post hoc pairwise comparisons with adjustment for multiple testing when the global test was significant. Duration of hospitalization, analyzed as a non-normally distributed continuous variable, is reported as median (IQR). Differences between years and between subgroups defined by sex, place of residence and comorbidity status were assessed using the Kruskal–Wallis test (global comparison) and the Mann–Whitney U test for selected pairwise comparisons, with correction for multiple testing.

To explore the association between comorbidities and demographic characteristics, age was compared between patients with and without a given comorbidity (cardiovascular disease, pulmonary disease, asthma), and the distributions of sex and place of residence were compared between these groups using the tests described above. Temporal trends in incidence rates were examined using linear regression models with calendar year entered as a continuous predictor; the slope coefficient and its *p*-value were used to assess the presence and direction of a linear trend, and the coefficient of determination (R^2^) was reported as a measure of model fit. Trends in the prevalence of comorbidities and in the proportions of women and of urban vs. rural residents among hospitalized patients over time were evaluated using tests for trend in proportions (Cochran–Armitage test) or logistic regression models with calendar year as a continuous covariate.

## 3. Results

**Figure 1:**
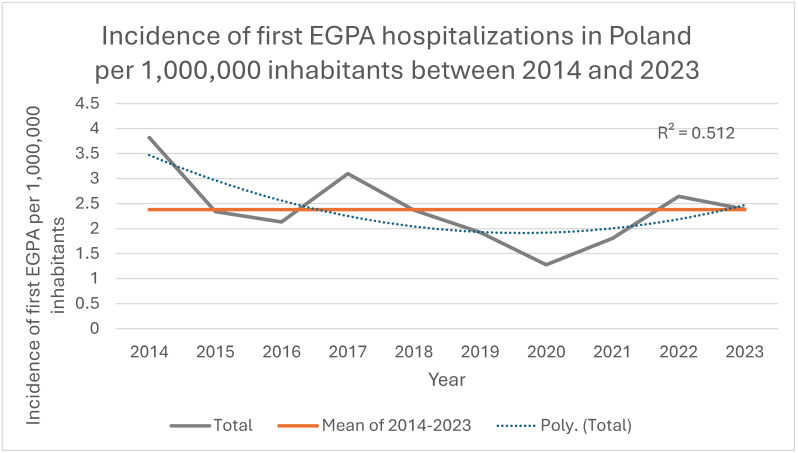
Incidence of first EGPA hospitalizations in Poland per 1,000,000 inhabitants, 2014–2023.

During 2014–2023, a total of 911 patients had a first EGPA-coded hospitalization in Poland, corresponding to an average annual incidence of 2.38 per 1,000,000 inhabitants. Annual incidence ranged from 1.28 per 1,000,000 in 2020 to 3.38 per 1,000,000 in 2023, with a significant decreasing trend between 2014 and 2019 (*p* < 0.001), while rates in 2021–2023 returned to levels similar to those observed in 2018 (Figure 1). Women accounted for 57.6% (*n* = 525) and men for 42.4% (*n* = 386) of first hospitalizations. Mean age at first hospitalization was 51.4 ± 16.2 years and did not differ by sex (*p* = 0.507) or across calendar years in the overall group (*p* = 0.523) or when women (*p* = 0.902) and men (*p* = 0.382) were analyzed separately. The median length of stay for first hospitalizations was 8 days (IQR 5–13) and was similar in women and men (*p* = 0.879), but became significantly shorter in 2023 compared with 2017 (*p* = 0.040) and 2018 (*p* = 0.049). These characteristics of first hospitalizations are summarized in Table 1.

**Figure 2:**
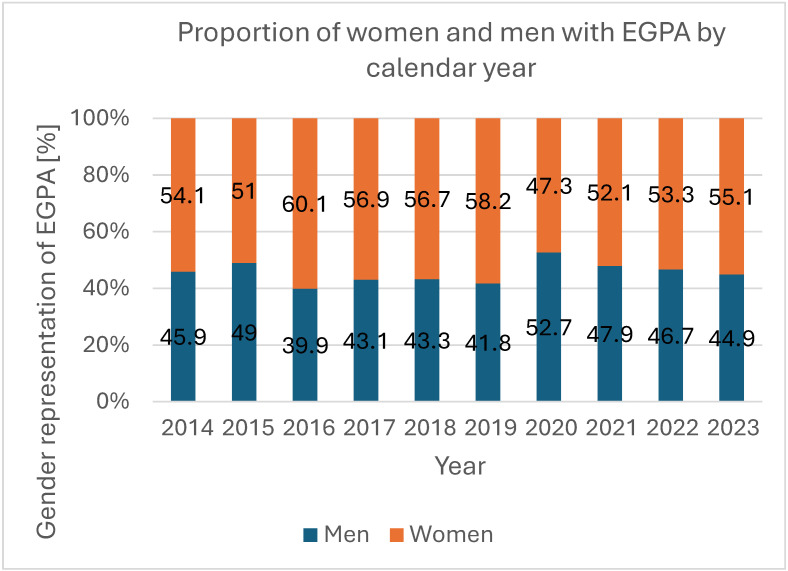
Proportions of women and men with EGPA by calendar year.

**Figure 3:**
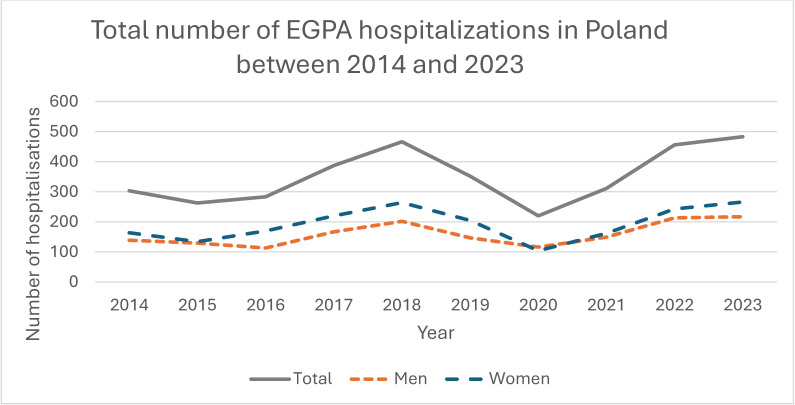
Total number of EGPA hospitalizations in Poland, 2014–2023.

**Figure 4:**
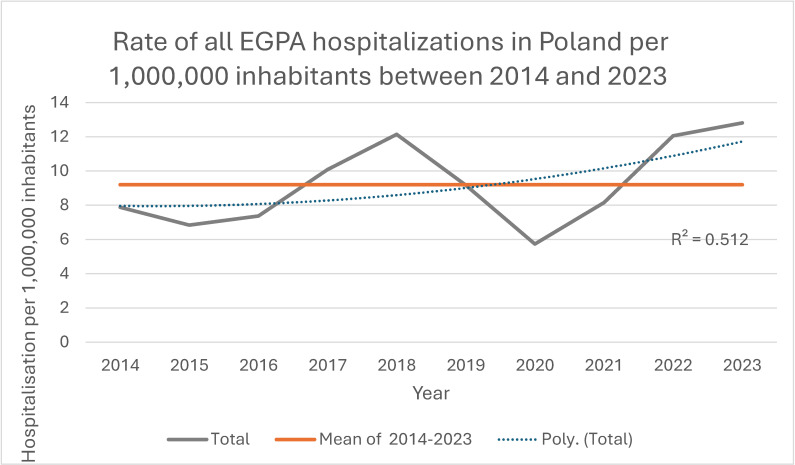
Rate of all EGPA hospitalizations in Poland per 1,000,000 inhabitants, 2014–2023.

**Figure 5:**
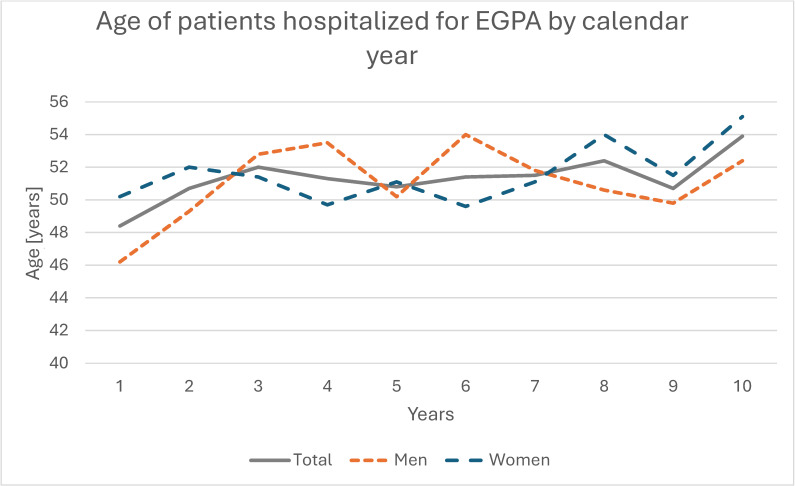
Age of patients hospitalized for EGPA by calendar year.

Overall, 3524 EGPA-coded hospitalizations were recorded during the study period; 54.8% (*n* = 1932) occurred in women and 45.2% (*n* = 1592) in men, with a significant female predominance (*p* < 0.001) and no significant change in sex distribution over time (*p* = 0.432; Figure 2, Table 2). The absolute number and population rate of EGPA hospitalizations showed variation across years (Figure 3 and Figure 4). Mean age for all EGPA hospitalizations was 51.4 ± 15.2 years and did not differ significantly between women and men (*p* = 0.247), but increased over years in the whole cohort (*p* < 0.001) and in women (*p* = 0.001) and men (*p* < 0.001) when analyzed separately. By 2023, men and women hospitalized with EGPA were on average 6.2 years (*p* = 0.009) and 4.9 years (*p* = 0.005) older, respectively, than in 2014 (Figure 5, Table 2). The median duration of stay for all EGPA hospitalizations was 5 days (IQR 3–8), similar in women and men (*p* = 0.587), but with a significant overall decrease across years (*p* < 0.001), reaching the shortest hospitalizations in 2023 in both sexes (Table 2).

**Figure 6:**
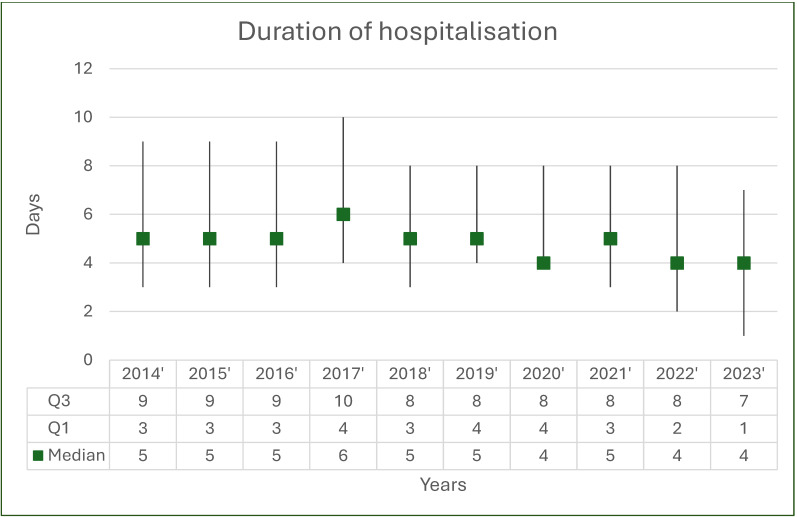
Duration of hospitalization.

**Figure 7:**
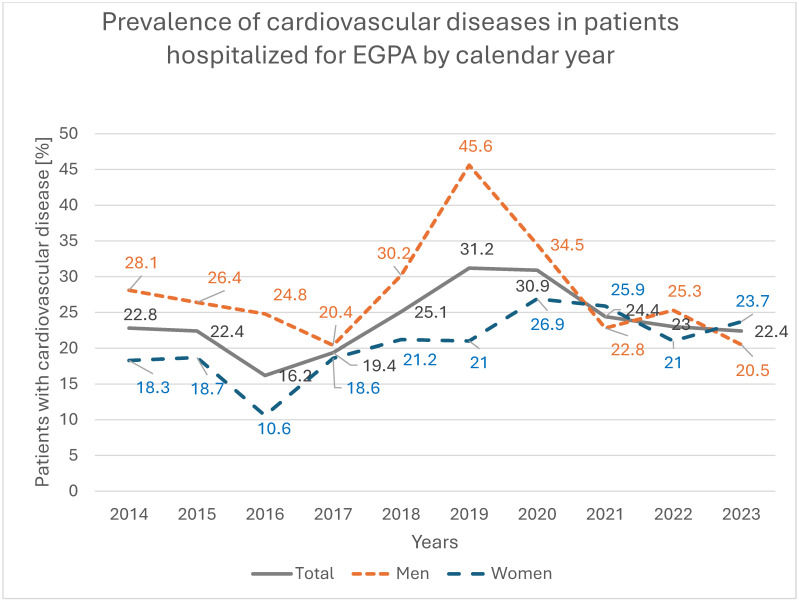
Prevalence of cardiovascular diseases in patients hospitalized for EGPA by calendar year.

**Figure 8:**
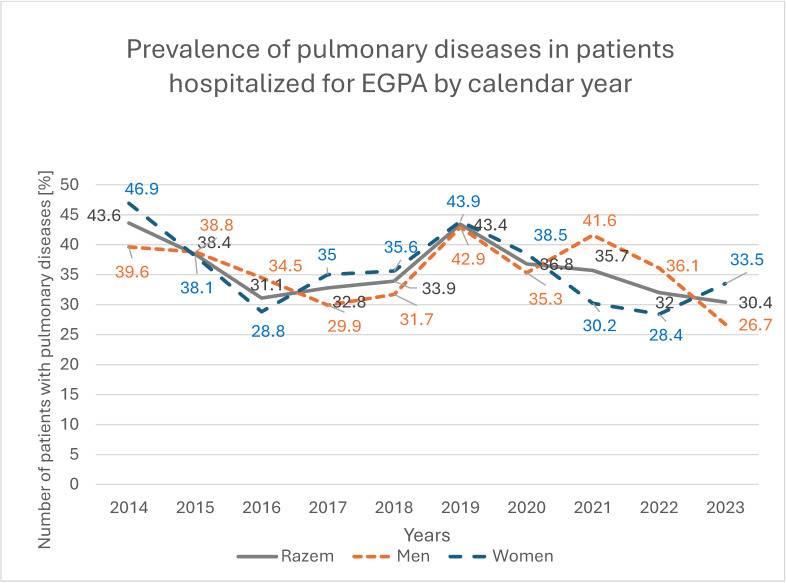
Prevalence of pulmonary diseases in patients hospitalized for EGPA by calendar year.

Cardiovascular disease was recorded in 833 patients (23.6%), more frequently in men than in women (27.4% vs. 20.5%; *p* < 0.001), with a significant increasing trend among hospitalized women (*p* = 0.004); by 2023, the prevalence of cardiovascular disease in women was 5.4 percentage points higher than in 2014 (Figure 6). Pulmonary diseases were present in 1244 patients (35.3%), with similar frequencies in women and men (35.5% vs. 35.1%; *p* = 0.832), but showed a significant decreasing trend among women, with a 13.4-percentage-point reduction between 2014 and 2023 (*p* = 0.009; Figure 7). Asthma was recorded in 1134 patients (32.3%), again with comparable prevalence in women and men (33.0% vs. 31.2%; *p* = 0.238), and a significant decreasing trend among women, in whom asthma prevalence decreased by 12.8 percentage points over the study period (*p* = 0.025; Figure 8).

**Figure 9:**
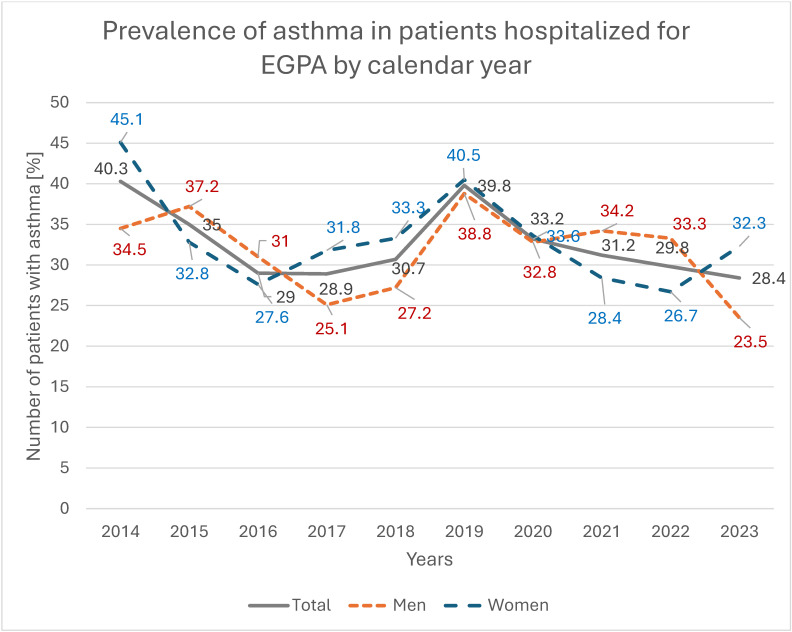
Prevalence of asthma in patients hospitalized for EGPA by calendar year.

Extended analyses of comorbidity patterns showed that cardiovascular and pulmonary disease clustered differently with age, sex and place of residence (Table 3 and Table 4). Cardiovascular comorbidities were significantly more frequent in rural than in urban residents (32.0% vs. 19.8%; *p* < 0.001), and patients with cardiovascular disease were older than those without (54.7 ± 14.1 vs. 50.3 ± 15.4 years; *p* < 0.001). Women with cardiovascular disease were older than men with cardiovascular disease (55.8 ± 14.3 vs. 53.7 ± 13.8 years; *p* = 0.036), whereas among patients with cardiovascular comorbidity, rural residents were younger than their urban counterparts (51.9 ± 13.6 vs. 56.8 ± 14.1 years; *p* < 0.001). In this rural subgroup, the youngest patients were hospitalized in 2017–2018 and the oldest in 2023, with statistically significant differences between these years (*p* = 0.020 and *p* = 0.022, respectively). In contrast, patients with pulmonary comorbidities were younger than those without pulmonary disease (48.2 ± 14.6 vs. 53.1 ± 15.3 years; *p* < 0.001), with no relevant differences between women and men (*p* = 0.473) or between urban and rural residents (*p* = 0.294) in terms of age. Among men with pulmonary disease, age increased over time, with those hospitalized in 2014 being significantly younger than those hospitalized in 2019 (*p* = 0.039), 2020 (*p* = 0.050) and 2023 (*p* = 0.015). These patterns indicate that cardiovascular comorbidity in EGPA is more frequent in older patients and in rural residents, whereas pulmonary comorbidity is associated with a younger age profile and shows less variation by sex and place of residence (Figure 9).

## 4. Discussion

In this nationwide, hospital-based study covering the years 2014–2023, we characterized the epidemiology and clinical profile of EGPA in Poland using discharge data for more than 900 patients with a first EGPA-coded hospitalization and over 3500 EGPA hospitalizations in total. We observed a low annual hospital-based incidence of approximately 2–3 first EGPA-coded hospitalizations per 1,000,000 inhabitants, with a decreasing trend between 2014 and 2019 and a marked disruption in 2020 coinciding with the COVID-19 pandemic, followed by a return to pre-pandemic levels thereafter. Over the study period the age of hospitalized patients increased and the duration of hospitalizations decreased, and we documented important changes in comorbidity patterns: cardiovascular disease became more frequent, particularly in older patients and those living in rural areas, whereas pulmonary comorbidities and asthma were recorded less often in women in later years. Together, these findings suggest an ageing, accumulating hospital-treated EGPA population in Poland and a possible shift in the clinical profile of patients seen in inpatient care.

Our hospital-based incidence estimates are broadly consistent with contemporary international data for EGPA. Recent systematic reviews and meta-analyses have reported pooled incidence around 1–2 cases per 1,000,000 person-years and prevalence in the range of 15–35 per 1,000,000 in mixed European and global cohorts [5,7]. In the UK, population data from 2005–2019 showed a relatively stable incidence of 2.3–4.0 per 1,000,000 person-years, accompanied by an increase in prevalence from 22.7 to 45.6 per 1,000,000 [8], while a nationwide Korean study reported a mean incidence of 1.2 per 1,000,000 and a rise in prevalence from 1.1 to 11.2 per 1,000,000 between 2007 and 2018 [9]. Our average annual incidence derived from first EGPA-coded hospitalizations falls within this range, although direct numerical comparison must be interpreted with caution, as many published analyses are based on larger or clinically richer registries, and our estimates are restricted to hospitalized patients. The most recent meta-analyses suggest that prevalence has increased over time despite relatively stable incidence, supporting the view that improved survival, earlier recognition and use of targeted therapies contribute substantially to the growing burden of EGPA [5,7,10].

Importantly, our study extends earlier Polish work based on the same national hospital morbidity database, which reported an average annual EGPA incidence of 1.5 per 1,000,000 (95% CI 1.2–1.8) and a point prevalence of 8.8 per 1,000,000 in 2013, with higher incidence in more urban regions [14]. Taken together, the earlier and current analyses indicate that hospital-based incidence of EGPA in Poland has remained low over the last two decades. The somewhat higher mean incidence in the current period compared with 2008–2013 may reflect improvements in coding of EGPA at the beginning of our observation period, whereas the decline in incidence from 2014 to 2019 and the return to pre-pandemic levels thereafter argue against an increase in disease occurrence. Our findings align with information from national cohorts and pooled analyses indicating relatively stable incidence, with changes in prevalence and hospitalizations caused more by case accumulation, survival and health-system factors than by underlying risk [5,7,8,9].

We observed a consistent female predominance among hospitalized EGPA patients (about 55% of cases) and a stable sex distribution across years. This is in line with several population-based studies, which have reported slightly higher incidence and prevalence in women than in men [8,9]. The mean age of hospitalized patients in our study (around 50 years) was similar in women and men but increased significantly over the decade, with men and women hospitalized in 2023 being approximately six and five years older, respectively, than those hospitalized in 2014. This ageing pattern likely reflects a combination of demographic ageing and improved survival and is compatible with the concept of a “maturing” EGPA cohort in which an increasing proportion of patients reach older ages and accumulate comorbidities and treatment-related side effects [5,7,10]. At the same time, the median duration of hospitalizations for EGPA shortened, suggesting more efficient inpatient management. However, prior studies underline that exposure to oral glucocorticosteroids remains high in EGPA [5,6,7,8], and our data highlight that this older, comorbid population will require careful balancing of disease control against treatment toxicity.

Our comorbidity analyses highlight the substantial cardiovascular and pulmonary burden accompanying EGPA. Cardiovascular disease was recorded in nearly one quarter of hospitalized patients and was more frequent in men than in women, more common in rural than in urban residents, and strongly associated with older age. This pattern aligns with evidence from clinical cohorts and imaging studies showing that cardiac involvement, ranging from subclinical myocarditis to chronic heart failure, is a major determinant of prognosis in EGPA [10,11,12,13]. Studies from referral centres have reported high rates of cardiac involvement and proposed screening strategies using echocardiography and cardiac MRI to detect early or silent disease [11,12,13]. Our nationwide findings support these recommendations and suggest that particular attention should be paid to cardiovascular risk assessment and cardiac screening in older EGPA patients and those living in rural areas.

In contrast, pulmonary comorbidities and asthma, although still common (around one third of hospitalized patients), showed a declining recorded prevalence over time among women. Given that asthma and upper airway disease are hallmark manifestations of EGPA and are reported in 70–90% of patients in clinical series [1,5,10], a plausible explanation for our observations is that more patients with predominantly airway disease are now managed in outpatient clinics rather than being hospitalized. During the period of our study, access to ANCA testing increased and care pathways for difficult-to-control asthma and chronic rhinosinusitis were implemented, as in other high-income countries [1,15]. The introduction of biologic agents, particularly mepolizumab and benralizumab, in EGPA and severe eosinophilic asthma has resulted in reduced complications, decreased oral glucocorticosteroid requirements and fewer hospitalizations [3,4,5,6]. These changes likely contribute to the decreasing recorded pulmonary disease and asthma in our hospital-based cohort.

The urban–rural differences we observed for cardiovascular, but not pulmonary, comorbidity are also noteworthy. Higher rates of cardiovascular disease among rural EGPA patients are consistent with epidemiological data showing that rural populations often have more adverse cardiovascular risk profiles and more limited access to specialized care than urban populations [2,11]. In our data, cardiovascular disease was both more prevalent and occurred at a younger age in rural residents, suggesting that EGPA management in these settings should incorporate proactive cardiovascular risk stratification and timely referral to cardiology services.

Taken together, these patterns have several implications for future research and clinical practice. First, the ageing, comorbid EGPA population we describe underscores the need for prospective studies evaluating structured cardiovascular screening algorithms and cardiometabolic risk-reduction strategies, particularly in older and rural patients. Second, the apparent shift in asthma-predominant disease towards outpatient management suggests that linkage of hospital data with outpatient and pharmacy records, as well as clinical EGPA registries, will be important to fully capture the impact of biologic therapies and to optimize long-term treatment pathways. Finally, our national, real-world estimates provide a benchmark against which future changes in therapeutic practice and healthcare organization in EGPA can be evaluated.

Our study has several strengths. It is based on a nationwide hospital morbidity database with mandatory reporting, covering inpatient care in Poland over a 10-year period. The large sample size and nationwide scope allowed us to assess temporal trends in hospital-based incidence, age structure, comorbidities and duration of hospitalizations, and to place these findings in the context of international literature [5,7,8,9,14].

However, several limitations should be acknowledged. First, our case definition relied on hospital discharge codes and could not be validated against clinical criteria such as the ACR/EULAR classification; therefore, misclassification of EGPA (both under- and over-diagnosis) is possible [1]. Second, we only had access to inpatient data. Patients with EGPA who were managed entirely in outpatient clinics, particularly those with milder or asthma-predominant disease, were not included, so our estimates reflect hospital-treated disease rather than the full EGPA population. Third, we defined “first” EGPA-coded hospitalizations from 2014 onwards and could not exclude EGPA-related admissions before the start of the observation period; some early-period “first” hospitalizations may therefore represent previously hospitalized patients, potentially increasing incidence in 2014 and complicating interpretation of early trends. Fourth, the registry does not contain anthropometric measurements or laboratory and imaging parameters (such as body mass index, blood pressure, eosinophil counts, biomarkers or cardiac imaging findings), nor information on specific treatments. As a result, we could not provide a detailed baseline table of anthropometric, haematological and biochemical characteristics or explore the effects of therapies on outcomes. In addition, sex- and age-specific population denominators were not consistently available, so we were unable to calculate fully age- and sex-standardised incidence rates to formally compare risk between women and men. Finally, because the registry contains only basic demographic information and ICD-10-coded diagnoses, without standardized indicators of disease activity, treatment intensity or clinical progression, we limited our analyses to descriptive statistics and simple group comparisons. More extensive multivariable or exploratory association analyses would have been prone to over-interpretation and an inflated risk of type I error and were therefore not performed. The COVID-19 pandemic, which clearly affected hospitalization patterns, represents an additional external factor that may have influenced our findings independently of underlying disease occurrence.

## 5. Conclusions

In this nationwide, hospital-based study from Poland, we provide robust estimates of hospital-based incidence and prevalence of EGPA and demonstrate that, over the last decade, EGPA hospitalizations have involved an increasingly older population with a substantial burden of cardiovascular and respiratory comorbidities. Hospital-based incidence remained low and broadly stable, while the number of hospitalized patients, their age and the accumulation of comorbidities increased and the length of hospital stay decreased, indicating a shift towards chronic, comorbidity-driven inpatient care rather than changes in underlying disease risk. Cardiovascular disease was particularly frequent in older patients and in those living in rural areas, whereas asthma and pulmonary comorbidities became less prominent over time, especially in women.

These findings have direct implications across rheumatology, pulmonology, cardiology and primary care. They support systematic cardiovascular risk assessment and targeted cardiac screening in EGPA—especially in older and rural patients—alongside continued attention to respiratory control and optimization of outpatient management to prevent avoidable hospitalizations. Together, our data underline the need for integrated, multidisciplinary care pathways to better capture, monitor and modify the long-term cardiovascular and respiratory burden in EGPA.

## Figures and Tables

**Table 1:** Number and age of patients with a first hospitalization for EGPA by calendar year.

		Total	2014	2015	2016	2017	2018	2019	2020	2021	2022	2023	*p*
Number of hospitalisations N (%)	Sum	911	149 (16.4)	90 (9.9)	81 (8.9)	118 (12.9)	92 (10.1)	74 (8.1)	48 (5.3)	70 (7.7)	100 (11.0)	89 (9.8)	
	Men	386 (42.4)	66 (44.3)	39 (43.3)	23 (28.4)	47 (39.8)	36 (39.1)	34 (45.9)	26 (54.2)	29 (41.4)	48 (48.0)	38 (42.7)	0.293
	Women	525 (57.6)	83 (55.7)	51 (56.7)	58 (71.6)	71 (60.2)	56 (60.9)	40 (54.1)	22 (45.8)	41 (58.6)	52 (52.0)	51 (57.3)	
Age (years)	Together	51.4 ± 16.2	49.3 ± 15.9	51.1 ± 16.0	51.8 ± 17.9	51.4 ± 16.3	51.1 ± 15.4	55.2 ± 14,4	49.1 ± 16.1	51.4 ± 16.7	52.1 ± 17.1	52.1 ± 16.0	0.523
	Men	50.9 ± 16.5	49.2 ± 16.5	50.5 ± 17.5	50.2 ± 17.7	53.2 ± 14.2	50.4 ± 14.9	57.9 ± 14.5	47.2 ± 17.1	48.9 ± 18.4	51.1 ± 17.7	50.3 ± 16.7	0.382
	Women	51.7 ± 16.0	49.3 ± 15.5	51.6 ± 15.0	52.5 ± 18.0	50.2 ± 17.6	51.6 ± 15.8	52.9 ± 14.1	51.3 ± 15.0	53.2 ± 15.4	53,0 ± 16.7	53.4 ± 15.6	0.902
Hospitalisation duration	Together	8 (5–13)	7 (4–12)	8 (5–12)	8 (5–13)	9 (6–13)	9 (6–14)	8 (5–11)	8 (6–13)	8 (5–13)	9 (5–12)	6 (4–10)	0.005
	Men	8 (5–13)	6 (4–12)	9 (5–14)	9 (6–13)	9 (5–14)	8 (6–13)	7 (6–14)	8 (6–15)	8 (4–12)	9 (5–13)	6 (4–10)	0.119
	Women	8 (5–12)	7 (4–12)	8 (5–11)	7 (5–14)	9 (6–13)	9 (6–15)	8 (5–10)	8 (6–10)	8 (5–13)	8 (5–12)	6 (4–11)	0.122

**Table 2:** Number and age of patients hospitalized for EGPA by calendar year.

		Total	2014	2015	2016	2017	2018	2019	2020	2021	2022	2023	*p*
Number of hospitalisations N (%)	Sum	3524	303 (8.6)	263 (7.5)	283 (8.0)	387 (11.0)	466 (13.2)	352 (10.0)	220 (6.2)	311 (8.8)	456 (12.9)	483 (13.7)	
	Men	1592 (45.2)	139 (45.9)	129 (49.1)	113 (39.9)	167 (43.1)	202 (43.3)	147 (41.8)	116 (52.7)	149 (47.9)	213 (46.7)	217 (44.9)	0.432
	Women	1932 (54.8)	164 (54.1)	134 (50.9)	170 (60.1)	220 (56.9)	264 (56.7)	205 (58.2)	104 (47.3)	162 (52.1)	243 (53.3)	266 (55.1)	
	Urban	2419 (68.7)	216 (71.3)	195 (74.1)	204 (72.1)	301 (78.4)	330 (70.8)	228 (64.8)	123 (55.9)	216 (69.4)	294 (64.5)	312 (64.6)	<0.001
	Rural	1102 (31.3)	87 (28.7)	68 (27.9)	79 (27.9)	83 (21.6)	136 (29.2)	124 (35.2)	97 (44.1)	95 (30.6)	162 (35.5)	171 (36.4)	
Age (years)	Sum	51.4 ± 15.2	48.4 ± 15.2	50.7 ± 14.4	52,0 ± 16.3	51.3 ± 15.2	50.8 ± 13.8	51.4 ± 14.7	51.5 ± 15.5	52.4 ± 15.7	50.7 ± 15.8	53.9 ± 15.5	<0.001
	Men	51.0 ± 15.3	46.2 ± 16.6	49.3 ± 15.2	52.8 ± 15.7	53.5 ± 14.3	50.2 ± 13.2	54.0 ± 13.9	51.8 ± 14.1	50.6 ± 15.8	49.8 ± 16.7	52.4 ± 15.5	<0.001
	Women	51.6 ± 15.2	50.2 ± 13.7	52.0 ± 13.4	51.5 ± 16.7	49.7 ± 15.6	51.2 ± 14.3	49.6 ± 15.0	51.1 ± 16.9	54.0 ± 15.4	51.5 ± 15.0	55.1 ± 15.3	0.001
	Urban	52.5 ± 15.3	48.1 ± 15.6	52.6 ± 13.2	53.2 ± 16.3	52.0 ± 15.3	51.9 ± 14.2	53.9 ± 14.5	55.5 ± 14.5	52.2 ± 15.9	52.2 ± 15.6	54.5 ± 16.1	<0.001
	Rural	48.9 ± 14.8	49.1 ± 14.2	45.0 ± 16.1	48.9 ± 16.0	48.3 ± 14.3	47.9 ± 12.4	46.9 ± 14.1	46.4 ± 15.2	52.9 ± 15.2	47.9 ± 16.0	52.8 ± 14.3	<0.001
Hospitalisation duration	Sum	5 (3–8)	5 (3–9)	5 (3–9)	5 (3–9)	6 (4–10)	5 (3–8)	5 (4–8)	4 (4–8)	5 (3–8)	4 (2–8)	4 (1–7)	<0.001
	Men	4 (3–8)	4 (3–8)	5 (3–9)	5 (3–8)	5 (3–9)	5 (3–8)	5 (4–8)	4 (2–8)	4 (2–7)	4 (2–8)	4 (1–7)	<0.001
	Women	5 (3–8)	5 (3–10)	6 (3–9)	5 (4–9)	6 (4–10)	5 (3–8)	5 (4–8)	5 (4–9)	5 (4–8)	4 (2–8)	4 (1–7)	<0.001
	Urban	5 (3–8)	5 (3–10)	5 (3–9)	5 (3–9)	5 (3–9)	5 (3–8)	5 (4–8)	5 (3–9)	4 (2–8)	4 (2–8)	4 (1–7)	<0.001
	Rural	5 (3–8)	4 (3–7)	5 (3–7)	5 (4–10)	8 (5–13)	5 (3–10)	5 (4–8)	4 (4–7)	5 (4–8)	4 (2–8)	4 (1–7)	<0.001

**Table 3:** Cardiological comorbidity among EGPA patients.

		Total	2014	2015	2016	2017	2018	2019	2020	2021	2022	2023	*p*
Number of cardiological diagnoses among EGPA patients N (%)	Sum	833 (23.6)	69 (22.8)	59 (22.4)	46 (16.2)	75 (19.4)	117 (25.1)	110 (31.2)	68 (30.9)	76 (24.4)	105 (23.0)	108 (22.4)	0.161
	Men	436 (27.4)	39 (28.1)	34 (26.4)	28 (24.8)	34 (20.4)	61 (30.2)	67 (45.6)	40 (34.5)	34 (22.8)	54 (25.3)	45 (20.7)	0.346
	Women	397 (20.5)	30 (18.3)	25 (18.7)	18 (10.6)	41 (18.6)	56 (21.2)	43 (21.0)	28 (26.9)	42 (25.9)	51 (21.0)	63 (23.7)	0.004
	Urban	478 (19.8)	48 (22.2)	41 (21.0)	28 (13.7)	47 (15.6)	65 (19.7)	61 (26.7)	27 (21.9)	44 (20.4)	55 (18.7)	62 (19.9)	0.684
	Rural	353 (32.0)	21 (24.1)	18 (26.5)	18 (22.8)	26 (31.3)	52 (38.2)	49 (39.5)	41 (42.3)	32 (33.7)	50 (30.9)	46 (26.9)	0.413
Age (years)	Without cardiac diseases	50.3 ± 15.4	46.6 ± 15.5	50.1 ± 14.3	50.9 ± 16.3	50.6 ± 15.8	50.6 ± 14.6	50.6 ± 15.4	50.1 ± 16.3	50.9 ± 15.0	49.7 ± 15.8	52.2 ± 15.1	0.016
	With cardiac diseases	54.7 ± 14.1	54.2 ± 12.3	52.6 ± 14.4	57.5 ± 15.5	54.2 ± 12.0	51.3 ± 11.2	53.3 ± 12.8	54.6 ± 13.0	56.8 ± 16.9	54.1 ± 15.6	59.8 ± 15.2	0.001
	Men with cardiac diseases	53.7 ± 13.8	53.6 ± 12.1	51.2 ± 14.5	57.4 ± 14.4	54.8 ± 11.3	49.7 ± 9.1	52.7 ± 12.2	52.2 ± 12.2	55.2 ± 18.2	55.8 ± 15.7	57.6 ± 17.3	0.101
	Women with cardiac diseases	55.8 ± 14.3	54.9 ± 12.9	54.4 ± 14.4	57.6 ± 17.5	53.6 ± 12.6	53.1 ± 12.9	54.3 ± 13.9	58.1 ± 13.4	58.1 ± 15.9	52.2 ± 15.5	61.3 ± 13.4	0.027
	With cardiac disease + urban	56.8 ± 14.1	54.8 ± 12.6	53.4 ± 15.0	57.9 ± 16.1	55.1 ± 10.3	53.6 ± 11.2	57.3 ± 11.4	61.0 ± 11.0	56.3 ± 17.5	58.3 ± 14.8	61.2 ± 17.7	0.055
	With cardiac disease + rural	51.9 ± 13.6	52.7 ± 12.0	50.8 ± 13.2	56.7 ± 15.0	51.7 ± 14.6	48.4 ± 10.6	48.4 ± 13.0	50.5 ± 12.6	57.5 ± 16.3	49.4 ± 15.3	57.9 ± 10.7	

**Table 4:** Pulmonological comorbidities among EGPA patients.

		Total	2014	2015	2016	2017	2018	2019	2020	2021	2022	2023	*p*
Number of pulmonary diagnoses among EGPA patients N (%)	Sum	1 244 (35.3)	132 (43.6)	101 (38.4)	88 (31.1)	127 (32.8)	158 (33.9)	153 (43.5)	81 (36.8)	111 (35.7)	146 (32.0)	147 (30.4)	0.005
	Men	559 (35.1)	55 (39.6)	50 (38.8)	39 (34.5)	50 (29.9)	64 (31.7)	63 (42.9)	41 (35.3)	62 (41.6)	77 (36.1)	58 (26.7)	0.207
	Women	685 (35.5)	77 (46.9)	51 (38.1)	49 (28.8)	77 (35.0)	94 (35.6)	90 (43.9)	40 (38.5)	49 (30.2)	69 (28.4)	89 (33.5)	0.009
	Urban	787 (32.5)	87 (40.3)	74 (37.9)	58 (28.4)	87 (28.9)	99 (30.0)	89 (39.0)	25 (20.3)	77 (35.6)	94 (32.0)	97 (31.1)	0.154
	Rural	456 (41.4)	45 (51.7)	27 (39.7)	30 (38.0)	39 (47.0)	59 (43.4)	64 (51.6)	56 (57.7)	34 (35.8)	52 (32.1)	50 (29.2)	<0.001
Age (years)	Without pulmonary disease	53.1 ± 15.3	50.3 ± 15.1	52.5 ± 13.8	53.3 ± 16.5	52.6 ± 15.6	52.0 ± 13.5	53.7 ± 15.0	54.0 ± 15.2	55.4 ± 16.0	51.8 ± 16.0	55.3 ± 15.4	0.008
	With pulmonary disease	48.2 ± 14.6	45.8 ± 15.0	47.7 ± 14.8	49.0 ± 15.6	48.6 ± 14.0	48.3 ± 14.2	48.4 ± 13.9	47.2 ± 15.0	46.9 ± 13.5	48.3 ± 15.1	50.5 ± 15.2	0.439
	Men with pulmonary disease	47.8 ± 14.7	42.0 ± 15.2	46.0 ± 14.1	45.6 ± 15.1	47.3 ± 14.1	47.4 ± 12.9	50.9 ± 12.2	51.8 ± 12.5	47.8 ± 14.4	47.4 ± 16.0	51.8 ± 17.1	0.013
	Women with pulmonary disease	48.4 ± 14.6	48.6 ± 14.3	49.4 ± 15.4	51.7 ± 15.5	49.5 ± 13.9	48.8 ± 15.1	46.7 ± 14.8	42.5 ± 16.2	45.8 ± 12.4	49.3 ± 14.1	49.7 ± 13.9	0.139
	With pulmonary disease + urban	48.5 ± 14.7	44.2 ± 15.7	47.9 ± 15.0	48.5 ± 15.3	48.6 ± 14.6	48.5 ± 15.7	49.2 ± 13.7	49.1 ± 12.9	47.9 ± 13.6	50.6 ± 14.4	50.3 ± 14.6	0.250
	With pulmonary disease + rural	47.6 ± 14.4	49.1 ± 13.0	47.2 ± 14.5	50.0 ± 16.2	48.3 ± 12.3	47.8 ± 11.5	47.4 ± 14.1	46.4 ± 15.9	44.7 ± 13.3	44.2 ± 15.7	51.1 ± 16.4	0.423

## Data Availability

No extra data available, all data is contained within the article.
